# Military psychiatry in India

**DOI:** 10.4103/0019-5545.69260

**Published:** 2010-01

**Authors:** H. R. A. Prabhu

**Affiliations:** Department of Psychiatry, AFMC, Pune, India

**Keywords:** Military Psychiatry, India, psychiatric centres

## Abstract

Military personnel, because of the unique nature of their duties and services, are likely to be under stress which at times has no parallel in civilian life. The stress of combat and service in extreme weather conditions often act as major stressors. The modern practices in military psychiatry had their beginning during the two World Wars, more particularly, the II^nd^ World War. The GHPU concept had the beginning in India with military hospitals having such establishments in the care of their clientele. As the nation gained independence, many of the military psychiatrists shifted to the civil stream and contributed immensely in the development of modern psychiatry in India. In the recent years military psychiatry has been given the status of a subspecialty chapter and the military psychiatrists have been regularly organizing CMEs and training programs for their members to prepare them to function in the special role of military psychiatrists.

## INTRODUCTION

The primary goal of Armed Forces of any country is to guard the nation against the external enemy. In the peace time role they also assist the civil administration in times of natural calamities at least in the initial part, because of their readiness to attend any such emergencies. The Armed Forces, because of the rigorous standards used in selection, ensure that only physically and mentally robust are recruited. The military training imparted ensures that the young raw recruit coming from a diverse socio-cultural environment gets transformed in to a battle worthy and disciplined soldier. Life in the Armed Forces exposes the personnel to such environments which many a times have no parallel in the civilian life- like living in isolated posts of extreme high altitudes, often being exposed to the life threatening combat environment. The family life of a soldier can get affected because of long periods of separation due to call of duty. Mental health issues assume importance in the backdrop of such issues. However, the working style of the Armed Forces ensures that the troops are on the constant watch of the immediate superiors almost on a daily basis so any deviation of behavior is likely to be noticed early and brought to the attention and taken care of. The military services have a well knit, time tested psychiatric service as part of their medical services to deal with the problems.

## THE PAST

### Pre World War II scenario

After the East India Company of the British established their base in India in the eighteenth century, as early as 1745, the first asylum for the care of British Soldiers, was established at Bombay as a part of the “Hospital” at Marine Yard. In 1795, the first hospital for mentally ill Indian sepoys was established in Monghyr, Bihar, which was subsequently disbanded in 1831.[1] In 1918 Col Owen Berkeley-Hill established European Mental Hospital at Ranchi and in 1922 Lt Col Lodge Patch at Lahore. The advent of World War II led to significant expansion of services for psychiatric services for the soldiers.[2]

### During World War II and the post-independent era

#### The establishment

At the outbreak of war there were only four specialists in psychiatry for the whole of troops in India. Because of their small number, most of the time the specialists recommended treatment to the patients with out seeing them and only on the basis of the reports sent to them.[2] The massive troop build-up, as war advanced, led to the establishment of service hospitals for the casualties and two specialists for mental diseases were authorized to the war hospitals. By August 1942 a pool of 10 specialists were created for peace hospitals which was increased to 31 by May 1943. By 1945 the strength of psychiatrists had increased to 86.[3,4] General duty medical officers (GDMO) with some training in psychiatry augmented the psychiatric services when the number of psychiatrists increased during war the senior psychiatrists were appointed as Advisors and they supervised the work of the other psychiatrists. While in the operational areas, particularly by 1944, there were psychiatrists posted up to Corps and Divisional level in Army and the psychiatrists established ‘Exhaustion centers’ to treat cases if battle exhaustion.

#### Training

Even though most GDMOs did not like to undergo training in psychiatry, those who were interested were exposed to training under senior qualified psychiatrists for three to four months after which they were posted as graded specialists. The training was mostly a hands-on experience of writing case histories, patient examination and diagnosing and suggesting treatment under the supervision of senior psychiatrist. The training used to be conducted at few centers like Calcutta and Lucknow. After many efforts it was found that training for a period of nine months to one year was considered the most efficient. Discussions on common medical problems and conferences were arranged.

#### Psychiatric centers

All the psychiatric centers of the military hospitals, both in war and peace, had been delivering services on the lines of General Hospital psychiatric units (GHPU). During war the psychiatric patients got treated in the psychiatric centers of different war time hospitals known as Indian Base General Hospitals (IBGH). There was separate authorization of beds for the Indian and British troops in different hospitals. The former, with 450 beds in three hospitals and the latter a total of 553 beds in five hospitals. Capt BL Chandorkar, who had served initially as MO, then as a trained psychiatrist in hospitals in Iraq and later at Alipore (Calcutta) and Dimapur has documented his war time experience as a psychiatrist.[5] After war he shifted to civil stream and finally served as Superintendent of mental hospitals of Thane and Yerwada.

A standard psychiatric ward had 25 beds and larger psychiatric centers had multiples of these. In July 1945, a 1000 bed psychiatric hospital was established at Jalahalli in Bangalore, which was disbanded later. In the large psychiatric centers one used to be the open ward where neurotic patients and quiet psychotic patients were kept. Psychotic patients mostly consisted of patients of schizophrenia and were kept in closed wards. Drugs used were hypnotics like bromides, chloral hydrate, phenobarbitone and paraldehyde. There was liberal use of paraldehyde orally or in injectable form to control aggression and one could always smell paraldehyde on entering such wards. Strait jackets and physical restraint to the bed were used too. Stable or less disturbed patients were used in making beds, cleaning ward utensils, fetching food etc. Recreation, as outdoor and indoor games, was generally available.

The psychotics were generally removed from service while neurotics who recovered from the symptoms were sent back to duty. Eventually most of them relapsed and were sent out of service. The staffing of the psychiatric centers was with mental health orderlies (MNO). Till 1943 there were about 100 British MNOs. Subsequently, Indian MNOs took over with which the environment in the psychiatric wards improved. The first ECT machine was brought to the country by Brig Bennet and it was used at Military Hospital Karachi. The machine was later used at Ranchi and Pune. By 1945 several military hospitals had ECT machines.[3]

#### Selection boards

An additional task that the psychiatrists were involved was in the selection of cadets for the Armed Forces. Each of the five selection boards - four for Army at Rawalpindi, Jabalpur, Calcutta, Bangalore and one for Navy at Lonavala had a psychiatrist on the panel and their work was supervised by an Advisor in Psychiatry at the Directorate of Selection Boards.[3]

#### The stalwarts

After leaving service, many of the military psychiatrists shifted to civil stream and contributed to the development of psychiatry in India. In 1947, Indian Psychiatric Society was established mainly due to the efforts of Major RB Davis.[5] Other notable names in this regard were Lt Col M R Vacha, Maj G A Bhagwat, Maj Vidyasagar, Capt B L Chandorkar, Maj KC Dube, Maj Je Dhunjeebhoy, Lt Col G R Parasuram. Col Kirpal Singh was the first senior psychiatrist of military services of Indian Origin in the post independent era. He joined military service in June 1941 and served till June, 1968. He was Professor and HOD Psychiatry at AFMC, Pune, from 1964-66 and later Senior Advisor in Psychiatry with Army Medical Corps as well as Defence Institute of Psychological Research at Delhi from 1966-68. He had a large number of original research papers in both national and international professional journals and was recipient of several fellowships and awards of professional bodies. His contributions to the development of psychiatry in India and military psychiatry in particular are immense.

## THE CONTEMPORARY

In the last three decades several military psychiatrists contributed to the growth of military psychiatry and many of them had been also active in the civil arena. They were Surg Rear Adm TB D’Netto, Brig SB Chatterjee, Brig RN Bhattacharya, Col GR Golecchha, Air Cmde IC Sethi, Maj Gen BP Singh, Surg Cmde MA Basit, Brig PK Chakraborty, Brig MB Pethe, Maj Gen PS Valdiya, Col DS Goel, Brig MSV Kama Raju, Brig Sudarsanan, Brig D Saldanha among many others. Most of them had been professors of psychiatry at the Armed Forces Medical College. Some of them had also been presidents of Indian Psychiatric society. Col DS Goel, after retirement, had a stint as a National Consultant at the Directorate General of Health Services, at Ministry of Health and Family Welfare, Government of India.

In 1997, 50 years after the inception of the Indian Psychiatric Society, Military Psychiatry was recognized as a subspecialty chapter. Ever since, the chapter has been organizing CMEs, seminars and presentations both independently as well as part of the Annual National conference of the IPS. On 21 September, 1997, Brig D Saldanha, then a psychiatrist at Military hospital Kirkee, Pune, organized the first CME on Military Psychiatry attended by large number of service as well as civil psychiatrists. Since then the CME programs have been held on a regular basis in various military stations. Armed Forces Medical College is the only medical college of the Armed Forces and a tri-service Category A training establishment, which undertakes undergraduate training for the future military doctors in psychiatry as well as post-graduate training in Psychiatry and military psychiatry.

Military psychiatrists of Army have been working in different and varied environments all over Indian subcontinent including border areas, areas affected by counter insurgency or those receiving soldiers in inhospitable high altitude terrains, jungles or desert areas. Those working in Navy and Air Force have been rendering their services for troops working at sea and in air environments. Original research has been conducted in to stress related to combat both in high intensity and low intensity counter insurgency operations, life in extreme high altitudes, life at sea, submarine environment and in flying personnel by the psychiatrists of the three services. Most such research in their areas of specialized work in the services is done under the auspices of the Armed Forces Research establishment and gets published in service medical journals.

## CONCLUSION

The foundation for Military Psychiatry in India was firmly laid during the few years of World War II. The contributions of service psychiatrists in the early years of inception of psychiatry in India have been substantial. With the unique nature of the working environments, the problems that the soldiers, sailors and air warriors face the work of military psychiatrists and the DRDO is of different kind and the unique research data brought are most of the time away from public domain due to security reasons. They find their application in the Armed Forces healthcare delivery. Military psychiatry, having evolved as an independent subspecialty, has come of age with lot of innovations and improvements in psychiatric services in the Armed Forces.
